# Spontaneous chronic epidural hematoma in the lumbar spine associated with Warfarin intake: a case report

**DOI:** 10.1186/s40064-016-3546-x

**Published:** 2016-10-21

**Authors:** Axel Sandvig, Håkan Jonsson

**Affiliations:** 1 Division of Neurosurgery and Clinical Neurophysiology, Department of Pharmacology and Clinical Neurosciences, Umeå University Hospital, Umeå, Sweden; 2 Spinal Unit, Department of Ortopedics, Umeå University Hospital, Umeå, Sweden; 3 Department of Neuroscience, Norwegian University of Science and Technology, Trondheim, Norway; 4 Spinal Unit, Department of Ortopedics, Uppsala University Hospital, Uppsala, Sweden

**Keywords:** Anticoagulant treatment, L5-root compression, Radiculating pain, MRI

## Abstract

**Introduction:**

Spontaneous spinal epidural hematomas are rare. However, in patients on anticoagulant treatment the risk may increase. Symptomatically patients may present with radiculopathy and even progressive neurological deficits.

**Case description:**

We present a case of a warfarin treated patient with left L5 radiculopathy. MRI was evaluated as showing a lumbar disc prolapse or synovial cyst at L4–L5 level. The patient was operated and an organized material was removed and analysed as a hematoma. No prolapsed disc or synovial cyst was found. The patient was neurologically restored following the operation.

**Discussion and Evaluation:**

This case illustrates how spontaneous epidural spinal hematomas can present with symptoms of radiculopathy and radiologically be misinterpreted as a protruding disc or cyst.

**Conclusion:**

Warfarin treated patients may have an increased risk of spontaneous spinal epidural hematomas.

## Introduction

Spontaneous spinal epidural hematomas occur infrequently (Mohammed et al. 2015). Epidural spinal hematomas located in the lumbar region are even more rare, particularly if they radiologically mimic a prolapsed disc or a discopathy. The venous epidural plexus is thought to be the source of the epidural hematoma. In most documented cases the hematoma is located posterior/posterolateral in the cervicothoracic or thoracolumbar junction (Narawong et al. 1988). The predilection for hematoma formation in these locations is hypothesised to be due to a junctional weakness in the epidural venous plexus.

The frequency of spinal epidural hematomas peaks at the age groups 15–20 and 47–75 years with males being affected more frequently than females (Narawong et al. 1988). In most cases the clinical presentation is acute with severe pain at the level of the hematoma progressing rapidly with sensory and motor deficits (Bennett et al. 2005; Le Coz et al. 1997; Groen 2004; Hang-ping et al. 2007; Sevizawa et al. 1995; Silber 1996). Bladder and bowel dysfunctions are common. Trauma, advancing age, anticoagulation, vascular malformations, Hemophilia, lumbar puncture, spinal anaesthesia, tumour, pregnancy, immune-mediated vasculitis and arterial hypertension are all documented risk factors for spinal epidural hematomas (Groen and Ponssen 1990; Narawong et al. 1988; Babayev and Ekşi 2016). Anti-coagulation treatment may account for 17 % of the reported cases to date (Silber 1996).

## Case report

We report on a 70-year old male with an atypical spontaneous epidural hematoma. The patient has signed an informed written consent to partake in this study His medical history included hypertension and atrial flutter, for which he received anti-coagulant treatment (Warfarin). He had undergone bilateral hip-replacement, the first on the right side and the second on his left side sixteen years later.

He presented with progressive lumbar radicular pain anterolaterally down the left leg and dorsomedially over the foot consistent with a left L5-radiculopathy. He also described periodic cramps in his left leg and foot, but no paresis or paresthesias. His bowel and bladder functions were normal. Pain relief occurred in recumbent position. The time from symptom-debut to operation was 6 months. He could not recollect having sustained any trauma to his back prior to the onset of his symptoms, nor during the period from symptom debut to operation.

Upon neurological examination the patient presented with a marked limp on his left foot. He was slightly palpation sore over his lumbar processus spinosi and paravertebral lumbar extensor muscles. He exhibited reduced lumbar flexion and reported increased radicular pain down his left leg and foot. He had no motor or sensory deficits. His sphincter function was normal. He developed a positive Lasuegé at 30° in his left leg, worsening with dorsiflexion of his left foot. His reflexes were 1+ symmetrically in his lower extremities. Babinski was negative bilaterally.

**Fig. 1:**
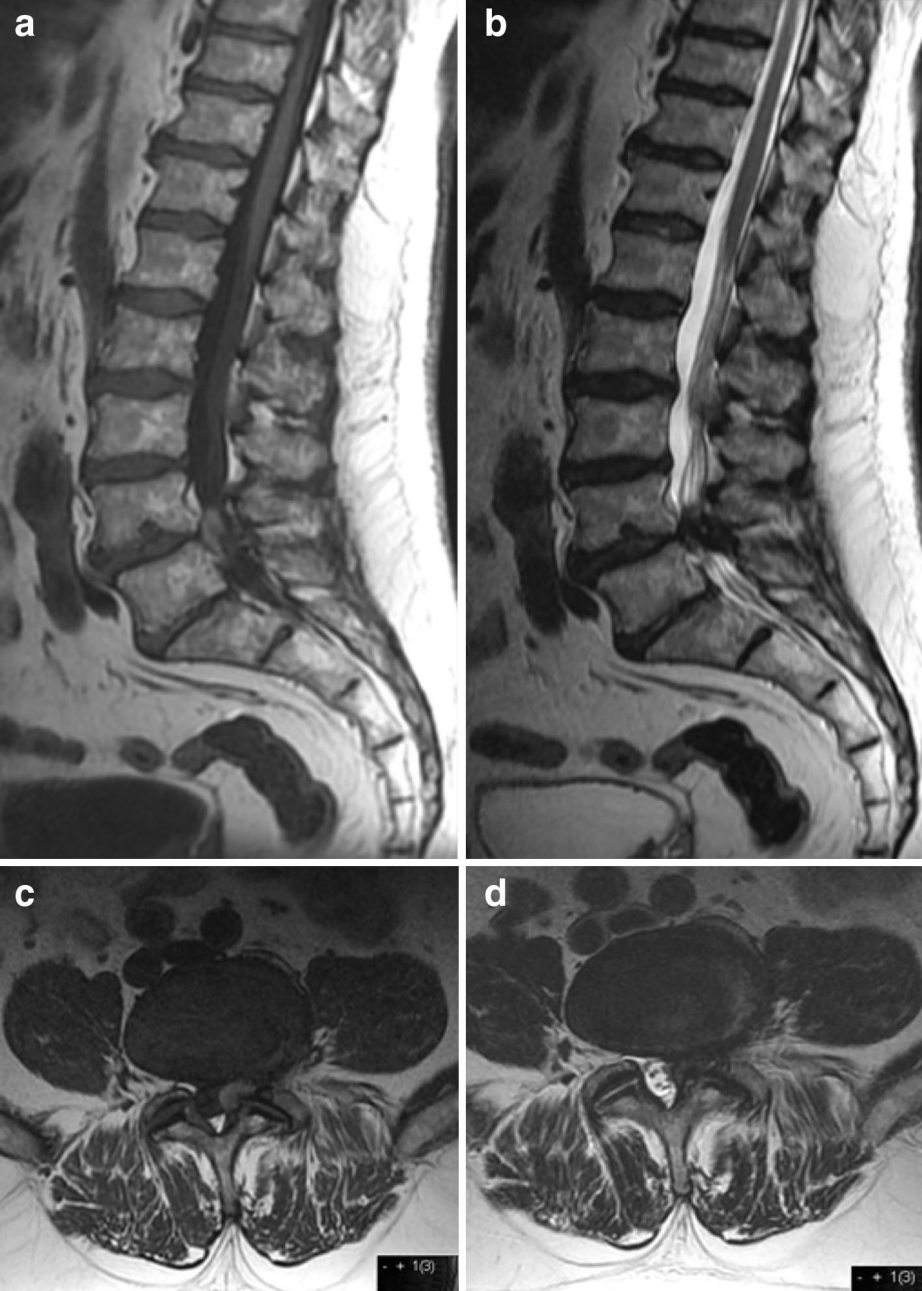
Sagital T1-weighted (**a**) and T2-weighted (**b**) axial T1-weighted image (**c**) and axial T2-weighted image (**d**) demonstrating a high signal intense epidural hematoma on the left side lumbar level L4–L5 and compressing the left L5-root

A lumbar MRI was performed and evaluated. Here, the radiological description supported the diagnosis of a prolapsed disc in lumbar level L4–L5. A differential diagnosis discussed was a synovial cyst on his left side at lumbar level L4–L5. Both T1- and T2-weighted images showed an extradural mass restricted to the dorsal space at lumbar level L4–L5 (Fig. 1). The axial images confirmed a mass, posterolateral to left-centred, at level L4–L5, which appeared to compress the left L5-root as it entered the intervertebral foramina (Fig. 1).

The laboratory work-up revealed an international normalized ratio (INR) within the range of 2–3. Prolonged thromboplastin time (PTT) and trombocyte numbers were within normal limits. He had normal temperature with CRP <10.

The patient underwent an elective operation planned as lumbar microdiscectomy at level L4–L5 6 months after symptoms began. During the operation a brownish material was encountered extradurally, which macroscopically appeared as an organised chronic hematoma and which could be followed laterally to where the left L5-root entered the foraminal canal. No prolapsed disc could be identified in the L4–L5 disc space.

**Fig. 2:**
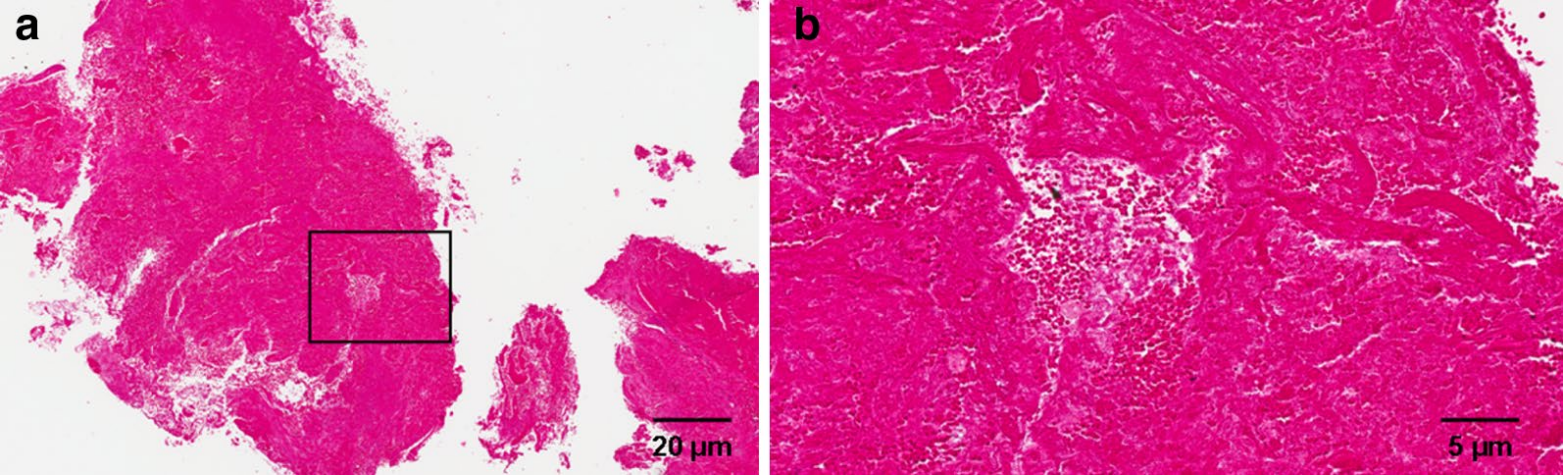
**a** Histopathological specimen of organised epidural hematoma and **b** higher magnification of boxed area in **a**

Histologic examination of the pathologic specimen confirmed the diagnosis of an organised chronic hematoma (Fig. 2). There was no evidence of a neoplasm or infection. Postoperatively he made an uneventful recovery with complete remission of his neurological symptoms including radicular pain.

## Discussion

The first case describing a spinal epidural hematoma was reported by Jackson in 1869 (Jackson 1869). Since then, more than 600 patients receiving surgical decompression of spontaneous spinal epidural hematomas (SSEH) have been reported (Groen and Ponssen 1990) and 112 pediatric cases (Yoneyama et al. 1998). In the last few years, several reports have been published describing the conservative treatment of SSEHs (Sagar and Hassan 2010; Riaz et al. 2008; Ross et al. 1987; Silber 1996; Sokolowski et al. 2008). The increase in the reported SSEHs is most likely due to increased use of MRI in establishing the radiological diagnosis (Oh and Lingaraj 2008).

Most SSEHs present with acute symptoms of severe pain with rapid signs of spinal cord and/or root compression. It is rare that patients present with slow progressive and chronic symptoms or intermittent relapsing radiculopathy that may mimic a prolapsed disc or spinal synovial cyst. In a review of the literature, Groen reported isolated root compression symptoms in 9 % of the patients (Groen 2004). In 67 % of patients, symptoms of spinal cord compression was present, cauda equina symptoms occurred in 2 % of cases while combined spinal cord and cauda equina symptoms were present in 5 % of the reported cases (Garzia et al. 1999).

It has been proposed that the internal vertebral venous plexus is the most likely source of SSEHs (Adamson et al. 2004; Hancock et al. 1997; Groen et al. 1997; Wait et al. 2005). Most hematomas are located posterior to posterolateral in the spinal canal.

The two regions of the spinal cord most predisposed to SSEHs are the cervicothoracic and thoracolumbar junctions. A purported weakness of the epidural venous plexus in these locations has been hypothesized as the reason for this preference (Adamson et al. 2004; Hancock et al. 1997; Jackson 1869; Wait et al. 2005).

In order to explain the more benign clinical course in some patients with SSEH, several mechanisms have been reported. Some argue that immediate replacement therapy in patients with coagulopathy prevents progression of the hematoma, allowing for relief of symptoms and neurological signs (Groen et al. 1997; Riaz et al. 2008). In contrast, Connelly et al. propose that spinal hematomas caused by coagulopathy can be managed conservatively because the hematoma remains liquid for a longer time than with normal clotting, allowing the liquid to dissipate in the spinal epidural space (Kebaish and Awad 2004). The findings in our case are contradictory to the proposal by Connelly et al. (Kebaish and Awad 2004). This may be due to serial bleeding adding more volume to the hematoma over time, which may lead to spinal cord and/or root compression symptoms. Inamasu et al. suggested that a liquefied hematoma may leak through the intervertebral foramen, which would lead to spontaneous decompression of the spinal cord (Wagner et al. 1996; Inamasu et al. 2000). If this was the case, a higher incidence of root compression symptoms would be expected. However, a previous report revealed that less than 10 % of patients diagnosed with SSEH had root symptoms (Oh and Lingaraj 2008). Furthermore, most hematomas are located posterior and not lateral in the spinal cord.

The proposal by Inamasu et al. is however interesting, with respect to our case report, in which L5-radiculopathy was a dominating presenting symptom. Furthermore, the MRI performed on our patient revealed a space occupying structure quite lateral on the left side at level L4–L5 which would likely cause compression of the left L5-root. Additionally, during the operation the organized hematoma was pursued laterally to where the left L5-root entered the intervertebral foramina. The patient also exhibited clinical symptoms of a left L5-root compression and had a positive Lasuegé on neurological examination.

Spreading of a hematoma may be possible in the acute phase following a bleed before blood clotting has occurred or in the subacute to chronic phases when the organised hematoma may, at least partially, become liquefied. Thus, patients on anticoagulation/platelet treatment or with an endogenous coagulopathy may experience a delayed clot formation allowing the liquefied hematoma to spread. However, the same mechanisms that delay clot formation may also promote further bleeding, which increases the space-occupying hematoma.

In a previous report in which patients with coagulopathy were excluded from the study (Kreppel et al. 2003), an analysis of SSEHs conservatively managed was compared to SSEHs surgically decompressed. The mean length of the hematoma along the spinal epidural space was significantly higher in those patients in which conservative management was applied, compared to the operated group (Oh and Lingaraj 2008). This finding may support the hypothesis that the liquefied hematoma may spread in the epidural space. An expanding hematoma may cause a release of the dural filaments crossing the epidural space, a process which is hypothesized to facilitate the spread of the haematoma, which results in a decompression of the dural sac (Kreppel et al. 2003).

## Conclusions

In conclusion, our case report emphasises the importance of being aware of the possibility of SSEHs, particularly in elderly patients with hypertension and on anticoagulant treatment. The history, neurological examination findings and radiological diagnostic work-up may mask this condition as a prolapsed disc or synovial cyst. The decision whether to operate or manage the patient conservatively should depend on the degree and type of symptoms and neurological findings.
